# Evaluation of the Costs and Consequences of Implementing an Optimization Process for Low-Complexity Emergency Care: The LINEA Program

**DOI:** 10.36469/001c.130031

**Published:** 2025-05-30

**Authors:** German Devia-Jaramillo, Nathalia Esmeral-Zuluaga, Juan Pablo Vargas-Gallo, Rafael Alfonso-Cristancho

**Affiliations:** 1 Fundación Santa Fe de Bogotá; 2 School of Medicine and Health Sciences Universidad del Rosario, Bogotá, Colombia; 3 Fundación Santa Fe de Bogotá, Colombia

**Keywords:** cost-consequence analysis, emergency department, overcrowding, fast-track

## Abstract

**Introduction:** Overcrowding is persistent in emergency departments (EDs) worldwide and can result in adverse patient outcomes and prolonged lengths of stay. Delays in care and unmet demand contribute to negative outcomes for patients awaiting treatment, including increased morbidity and mortality, prolonged hospital stays, and overall lower quality of medical care. Overcrowding in EDs not only diminishes patient satisfaction with the entire hospitalization experience, beyond the ED, but also significantly increases healthcare costs and contributes to a rise in medical errors. Therefore, developing strategies that optimize the limited resources available for emergency patient care, especially for those with low-complexity emergencies, is crucial. **Objective:** To evaluate whether implementing a specific care strategy for patients with low-complexity emergencies can effectively reduce costs and improve clinical outcomes and patient-reported experiences compared with standard care practices. **Methods:** A cost-consequence model was employed to separately evaluate the costs and outcomes of each alternative. The cost and outcome analyses were applied to healthcare services using the database of a tertiary-level ED, analyzed from the perspective of the healthcare service provider over a 2-year time horizon. To assess the perspective of the healthcare provider institution, the cost-consequence analysis was conducted using a decision tree model. **Results:** The study included 43 268 patients. No significant differences were found in demographic variables between groups. A significant difference was found in total length of stay in minutes between groups: minimum (median interquartile range [IQR]), 534 (456-644) vs 494 (364-719) (*P* < .001). In addition, there was an improvement in the NPS value from 44 to 53 throughout the ED, with 0.005% mortality in the study group and 0.07 in the control group (*P* < .001). Finally, a significant difference was documented in the mean billing per patient, with a median (IQR) of Col255 903(Col151 108-Col658 585)vsthecomparisongroupandCol283 922 (Col125 998−Col776 097) (*P* < .018). **Conclusion:** The implementation of a specialized unit for the care of patients with low-complexity emergencies within the ED has proven effective in improving total patient length of stay. This significantly contributes to reducing overcrowding, decreasing mortality, and reducing unmet demand. As a result, there is an overall improvement in user satisfaction within the ED.

## INTRODUCTION

Overcrowding remains a persistent challenge in emergency services worldwide.^1^ This situation can result in adverse patient outcomes and prolonged lengths of stay (LOS).^2^ Extended hospital stays are associated with challenges in accessing suitable hospital beds, missed treatment opportunities due to ambulance diversions, and instances of patients being left without being seen.^3^ Delays in care and unmet demand have been documented as factors contributing to negative outcomes for patients awaiting treatment, including increased morbidity and mortality, prolonged hospital stays, and overall lower quality of medical care. Overcrowding in emergency departments (EDs) not only diminishes patient satisfaction with the entire hospitalization experience, beyond just the ED segment,^4^ but also significantly increases healthcare costs^5^ and contributes to a rise in medical errors.^6^

According to the Joint Commission on Accreditation of Healthcare Organizations, 50% of sentinel events occur in EDs, with one-third of these attributed to overcrowding.^2^ Adverse outcomes associated with overcrowding are independent of the patients’ triage classification. Delays in care are linked to increased mortality, regardless of the initial triage classification.^7^ Therefore, it is crucial to develop strategies that optimize the limited resources available for emergency patient care, especially for those presenting with low-complexity emergencies. This study aims to evaluate whether implementing a targeted care strategy for these patients can effectively reduce costs and enhance both clinical outcomes and patient-reported experiences when compared to standard care practices. Furthermore, the study seeks to assess the strategy’s impact on decision-making time, alleviate overcrowding, and improve overall user experience.

## METHODS

### Study Design

**Figure 1.**
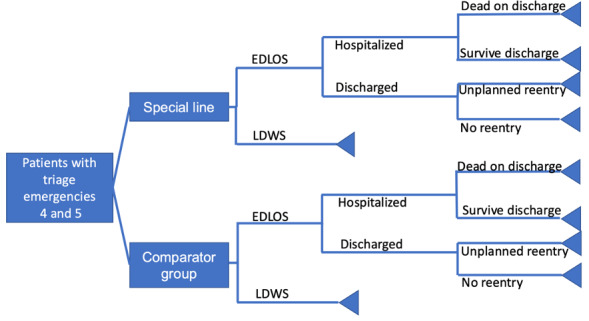
Decision Tree Model Abbreviations: EDLOS, emergency department length of stay; LDWS, left without being seen.

A cost-consequence model was employed to separately evaluate the costs and outcomes of each alternative. The cost and outcome analysis were applied to healthcare services using the database of a tertiary-level ED, analyzed from the perspective of the healthcare service provider over a two-year time horizon. To assess the perspective of the healthcare provider institution, the cost-consequence analysis was conducted using a decision tree model (**Figure 1**).

### Population

The study utilized databases from the ED of the Hospital Universitario Fundación Santa Fe, a high-complexity institution that handles approximately 60 000 adult emergencies annually. Of these cases, 56% are classified as low complexity, corresponding to patients categorized as triage levels 4 and 5 according to the Emergency Severity Index triage classification system. These patients with less serious emergencies do not represent an obvious risk to life or loss of limb or organ; however, there are risks of complications or sequelae of the disease or injury if they do not receive the corresponding attention. These patients could wait around 240 minutes to be seen in the ED.^8^

### Inclusion Criteria

The study included adult patients (>18 years old), evaluated in the ED of the Hospital Universitario Fundación Santa Fé, classified as low-complexity emergencies, specifically triage levels 4 and 5. Additionally, only patients assessed under the “special line” care protocol between February 2019 and February 2020 were included. For the comparator group, patients with similar characteristics who were attended to betweem February 2018 and January 2019 were included. Exclusion criteria comprised pregnant patients, pediatric populations, orthopedic patients, and those with incomplete data.

### Methodology

The study utilized institutional databases from before and after the implementation of the patient care protocol under evaluation, covering the period from February 2018 to February 2020. This study also analyzed ED stay times and patient-reported outcomes. However, these analyses were not conducted prospectively due to modifications in the emergency services caused by the COVID-19 pandemic, which have not yet returned to pre-pandemic operations. Consequently, it was not feasible to measure quality of life or derive utilities for each patient to conduct a traditional economic evaluation. Therefore, a cost-consequence analysis was considered appropriate for this study.

Patients who attended in 2019 were designated as the intervention group, while those evaluated in 2018 served as the comparison group. The intervention group, referred to as the “special line,” was implemented from Monday to Friday at 10 AM and 7 PM for patients with low-complexity emergencies, specifically triage levels 4 and 5. These schedules were initially implemented because this was a new intervention and we wanted to avoid too much impact on the control of the ED. Since this change could negatively impact the ED, stricter monitoring was necessary, which was more feasible on weekdays than on weekends. Additionally, the protocol was initially established for certain specific insurance providers. We used these same criteria to select patients for the control group.

### Intervention Protocol

The strategy known as the “special line” is a method that combines a fast-track strategy with an Acute Medical Unit. This tool is designed for the care of patients with low-complexity emergencies. Initially, a nurse identifies patients presenting with triage level 4 and 5 emergencies using the ESI triage classification.^8^ Subsequently, the patient is directed to an area within the ED called the special line.

In this area, a specific team of nurses and doctors is responsible for patient management, from the initial assessment to their placement in the ED. The diagnostic and therapeutic process is guided by institutional protocols, and there are no differences in the treatment of other patients.

In this study, no additional staff were hired; the study was conducted with the available ED personnel.

The special line is equipped with two consultation rooms that meet national standards for low-complexity emergency care. Additionally, there is a space for laboratory sample collection. The transport routes for samples and transfers to diagnostic imaging are the same as those used for patients with higher-complexity emergencies.

The support staff and consultation processes are consistent with those of the entire ED.

### Sample Size

All patients who met the inclusion criteria during the study period were included in the analyses. As this is a retrospective observational study, no sample size calculation was performed.

### Measured Outcomes

The following outcomes were measured in both the intervention group and the comparison group:

- **Percentage of patients leaving without being seen (LWBS):** This refers to the patients who leave the ED without having been seen by a physician, indicating unmet demand.^9^
- **Unscheduled readmissions**: Defined as an unplanned return visit to the ED within 72 hours after a patient has been discharged.
- **Emergency department length of stay** (EDLOS): This is a condition resulting from overcrowding in the ED. The term EDLOS refers to the total LOS in the ED.^2^
- **Time to consultation**: For this study, time to consultation is defined as the time measured between the nurse’s triage assessment and the start of the physician’s care in the emergency consultation.
- **Time to disposition**: This is measured from the time the physician’s consultation begins to the moment a definitive decision is made regarding the patient’s care, such as discharge, hospitalization, or surgery.
- **Mortality**: This includes patients who presented to the ED, were classified as triage levels 4 or 5, and were certified deceased by institutional personnel due to hospital care associated with the emergency consultation during the study year.
- **Billing costs**: The total costs billed to insurers for services provided in the ED were recorded.
- **Net Promoter Score (NPS) value**: The NPS Index is a method used by the institution to evaluate patient experience during their stay in the ED. The scale measures the difference between detractors and promoters, with a score of +100 indicating all promoters who would recommend the institution, and a score of -100 indicating all are detractors.^10^

### Data Analysis

The intervention’s impact was assessed by comparing two distinct periods:

1. **Before the intervention**: This period includes patients who were attended to in 2018. It serves as the baseline for evaluating the effectiveness of the intervention.
2. **After the intervention**: This period includes patients who were attended to in 2019. It represents the time frame after the implementation of the intervention strategy. To determine their comparability, a comparison was made of the demographic variables collected, age, number of patients, gender, admission diagnosis, number of triage 4 cases, and number of triage 5 cases, depending on whether they are quantitative or categorical variables. The differences were established by *t*-test (if the variable has a normal distribution), Wilcoxon test (if it does not have a normal distribution), or χ^2^ test for categorical variables. For categorical variables, frequency distribution was performed; for continuous variables, central tendency and dispersion measurements were performed according to the type of distribution (mean and standard deviation or median and interquartile ranges (IQR) for normal or non-normal distribution, respectively). Subsequently, statistical significance was established in the mean differences in the values of LOS, waiting time, and level of satisfaction using the *t*-test for variables with a normal distribution or Wilcoxon test for variables with a non-normal distribution. In the case of comparison of categorical variables, these were evaluated using the χ^2^ test. The analyses were performed using R version 1.4.

## RESULTS

**Figure 2.**
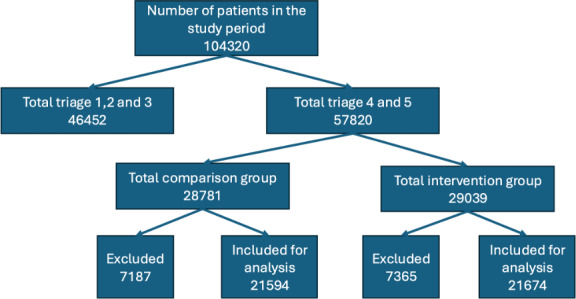
Patient Selection Algorithm

During the comparison year, a total of 51 160 emergency cases were attended, whereas, in the intervention year, this number increased to 53 112. For patients classified as triage 4 and 5 emergencies, 21674 were included in the analyses for the intervention year, compared with 21 594 in the comparison year. **Figure 2** provides a detailed overview of the patient flow admitted for the analyses, ensuring a clear understanding of the dataset used to assess the impact of the intervention.

No significant differences were found in demographic variables between groups (**Table 1**).

**Table 1.** Patient Characteristics from the Comparison and Intervention Groups

	**Comparison Group**	**Intervention Group**	***P* Value**
n (%)	21 594 (100)	21 674 (100)	
Male	9609 (44.5)	9710 (44.8)	.326
Female	11 985 (55.5)	11964 (55.2)	
Age, median (SD)	51.76 (19.53)	50.5 (17.19)	.052
Medical insurance, n (%)			.221
Colmedica	5852 (27.1)	5245 (24.2)	
Colsanitas S.A.	2289 (10.6)	2969 (13.7)	
Allianz	2267 (10.5)	2644 (12.2)	
Colpatria	1814 (8.4)	1799 (8.3)	
Medplus	1296 (6.0)	1279 (5.9)	
Pago directo	1188 (5.5)	1452 (6.7)	
Int. Comp.	929 (4.3)	1040 (4.8)	
Diagnosis, n (%)			
Infectious	7795 (36.1)	7899 (36.4)	.641
Cardiovascular	1533 (7.1)	1669 (7.7)	.095
Trauma	2440 (11.3)	2146 (9.9)	.001
Surgical	1209 (5.6)	1214 (5.6)	.933
Neurological	2267 (10.5)	2081 (9.6)	.041
Skin	972 (4.5)	975 (4.5)	.940
Metabolic	907 (4.2)	780 (3.6)	.024
Toxic	777 (3.6)	780 (3.6)	1.000
Psychiatric	799 (3.7)	824 (3.8)	.702
Cancer	1058 (4.9)	737 (3.4)	<.001

Abbreviation: Int. Comp, international insurance company.

Upon comparing the care times, a significant difference was observed in both the consultation time and the total ED stay of the evaluated patients (**Table 2**).

**Table 2.** Comparison of Times Between the 2 Groups

**Variable**	**Comparison Group**	**Intervention Group**	***P* Value**
Time to consultation (min), median (IQR)	272 (178-295)	238 (89-302)	<.001
Time to definition (min), median (IQR)	509 (172-802)	477 (145.5-803)	<.001
EDLOS (min), median (IQR)	534 (456-644)	494 (364-719)	<.001

Abbreviations: EDLOS, emergency department length of stay; IQR, interquartile range.

The unmet demand (LWBS) and the overall NPS score across the ED during the preintervention and postintervention periods showed a reduction in unmet demand, with a median decrease from 2.350 (range, 1.900-2.900) in the comparison period to 1.850 (range, 1.650-2.375) in the intervention period (*P =* .379). Regarding the NPS value, it was documented that the average score for this index across the entire ED increased from 44 (SD = 7.471) during the

comparison period to 56.3 (SD = 8.906) in the intervention period. In terms of the negative outcomes evaluated, it was found that the comparison group had 4992 revisits (23.1%), compared with the intervention group, which had 5996 revisits (27.7%). A significant association was found for the intervention group with an odds ratio (OR) of 1.261 (95% CI, 1.207-1.317; *P <* .001).

Regarding mortality, the comparison group recorded fifteen deaths (0.07%), while the intervention group documented only one death (0.005%). This demonstrated a protective association for the intervention group with an odds ratio (OR) of 0.075 (95% CI, 0.003-0.371; *P <* 001).

In terms of billing expenses, an investment of Col.$200 000 000 was made for the construction of the fast-track area. No additional costs were incurred for personnel, as there was a redistribution of functions. A significant increase was documented in the annual billing costs between the groups, with Col.$34 042 686 156 for the comparison group and Col.$37 743 105 027 for the intervention group. When comparing billing expenses per patient, a significant difference was documented between the groups, with a median (IQR) of Col.$255 903 (Col.$151 108-$658 585) for the comparison group and Col.$283 922 (Col.$125 998-$776 097) for the intervention group (*P =* .018). Subsequently, we carried out a sensitivity analysis that was applied to the billing costs minus 20%, which was considered an approximation of the production costs (**Table 3**).

**Table 3.** Cost Analysis

**Variable**	**Comparison Group**	**Intervention Group**	***P* Value**
n	21 594	21 674	
Total investment cost, Col$	0	200 000 000	
Annual billing cost, Col$	34 042 686 156	37 743 105 027	
Billing charges per patient, median (IQR), Col$	255 903 (151 108-658 585)	283 922 (125 998-776 097)	.018
Production costs per patient, median (IQR), Col$	51 181 (30 222-131 717)	56 784 (25 200-155 219)	.018
Sensitivity analysis per patient, median (IQR), Col$	204 722 (12 886-533 868)	227 138 (100 798-620 878)	
Direct costs (monthly), Col$			.018
Nursing assistants, mean (SD)	12 415 800 (0)	12 415 800 (0)	
Nurses, mean (SD)	29 997 266 (0)	29 997 266 (0)	
Specialist physician, mean (SD)	50 748 754 (0)	50 748 754 (0)	
General practitioners, mean (SD)	11 376 239 (0)	11 376 239 (0)	
Support staff, mean (SD)	6 500 090 (0)	6 500 090 (0)	
Indirect costs per month, Col$			.018
Administrative assistant	3 434 204 (0)	3 605 914 (0)	
General services	4 573 810 (0)	4 802 500 (0)	
Consulting rooms	0	18 000 000 (0)	
Observation stretchers	75 460 000 (0)	75 460 000 (0)	ND

Abbreviations: Col$, Colombian pesos; IQR, interquartile range.

Finally, by carrying out indicators related to investment and viability, it could be determined that the return on investment was positive (18.7); likewise, the net present value was positive.

## DISCUSSION

This study assessed the effectiveness of implementing a care strategy for low-complexity patients in a general ED by comparing variables before and after the intervention. Initially, it was determined that, despite being a retrospective study, the groups were comparable. No significant differences were found in terms of patient gender, age, diagnoses, or insurance providers.

The comparison of patient stay times resulting from using different protocols demonstrated that implementing the “line” strategy significantly reduced the total length of stay in the ED. The findings are consistent with other research showing that fast-track strategies effectively reduce patient stay times in emergency settings. Sánchez et al demonstrated a significant reduction in EDLOS from 285 (±45) minutes to 258 (±40) minutes (*P* < .001) with the implementation of a fast-track strategy in medium-complexity EDs.^11^ This further supports the effectiveness of fast-track strategies in reducing patient stay times in emergency settings.

Similarly, Considine et al demonstrated the effectiveness of the fast-track strategy in significantly reducing the LOS for patients in EDs.^12^ Another study conducted by nursing staff showed that patients with minor injuries and illnesses who were referred to a fast-track area experienced a reduction in wait time from 56 to 30 minutes, and a decrease in EDLOS from 57 to 34 minutes.^13^ Additionally, referring patients with minor traumatic injuries to fast-track zones resulted in a 30% reduction in the time to first contact with a physician.^14^

The evaluated care strategy demonstrated a reduction in unmet demand following the intervention, although the data was not statistically significant. This finding is noteworthy as it aligns with the work reported by Darrab et al, where the implementation of a fast-track protocol resulted in a decrease in the total number of patients leaving without being seen, from 5% to 2%.^15^

The overall NPS of the ED increased following the implementation of the “line” care protocol. This is particularly interesting as it highlights that low-complexity emergencies make up a significant portion of the patients seen in the ED. Their satisfaction positively influences the perception of the overall quality of care provided by the entire department. Leslie et al demonstrated that the NPS is useful in determining the performance of health services.^16^ Although there are not many publications that evaluate user satisfaction with emergency interventions using the NPS value, the study by Vu et al demonstrated that by adjusting the antibiotic administration program for patients with appendicitis in emergency settings, the NPS for these patients increased significantly from 26.0 to 78.3.^17^ A study conducted in the United Kingdom demonstrated that the implementation of a fast-track unit for surgical pathologies in emergency settings significantly improved the NPS value for users, increasing it from 2.2 to 64.1.^18^

In this study, an increase in the number of follow-up visits to the ED was documented. However, these follow-up visits did not impact the mortality outcome, as a significant reduction in mortality was demonstrated during the intervention year compared with the previous year. A study by Navarro et al highlighted that, despite the rise in follow-up visits, the implementation of effective interventions in emergency settings can lead to a notable decrease in mortality rates.^18^ Despite the high initial investment required for the creation of the fast-track area, billing expenses increased significantly when comparing one year to the next. Additionally, these expenses are likely to continue rising year after year, without the need for major new adjustments. This suggests that the initial setup costs can be offset by the increased efficiency and throughput of the fast-track system, leading to higher revenue generation over time.

### Limitations

One of the key limitations of this study is that it was conducted in a single institution, which restricts the ability to generalize the findings to other settings. While the results may apply to institutions with similar characteristics, it is crucial to consider that variations in infrastructure, resources, and protocols can impact the outcomes. The main bias in our work could be the validity of the comparison between the groups. However, the demographic characteristics of the two populations were compared, and no major significant differences between the groups were found. Therefore, we believe the groups were susceptible to the analysis we performed and the results could be reliable.

## CONCLUSION

The implementation of a specialized unit for the care of patients with low-complexity emergencies within the ED has proven effective in improving the total LOS for patients. This significantly contributes to reducing overcrowding, decreases mortality and reduces unmet demand. As a result, there is an overall improvement in user satisfaction within the ED.

### Declarations

The study was conducted at the Hospital Universitario Fundación Santa Fé de Bogotá, Colombia, and was approved by the institution’s research and ethics committee (No. CCEI-15670-2023). This study was performed in accordance with the ethical standards as laid down in the 1964 Declaration of Helsinki and its later amendments. The researchers did not expose the patients to biological, psychological, or social risks. Therefore, the ethics committee approved the waiver of informed consent. This research is classified within the “no risk” category. Access to research instruments was limited only to investigators according to Article 8 of Resolution 008430/1993 by the Colombian Ministry of Health.

### Disclosures

The authors disclose no competing interests.

### Availability of Data and Materials

The datasets used and/or analyzed during the current study are available from the corresponding author upon reasonable request.
